# A direct method for imaging gradient levels of retinal hypoxia in a model of retinopathy of prematurity (ROP)

**DOI:** 10.21203/rs.3.rs-7247191/v1

**Published:** 2025-08-21

**Authors:** MD Imam Uddin, Sara Jamal, John S. Penn

**Affiliations:** Vanderbilt University School of Medicine; Vanderbilt University School of Medicine; Vanderbilt University School of Medicine

**Keywords:** Retinopathy of prematurity, ROP, molecular imaging, retinal hypoxia, HYPOX-4, optical imaging, fluorescence imaging

## Abstract

**Background:**

Retinal hypoxia may contribute to the development of preretinal neovascularization in patients with retinopathy of prematurity (ROP). Ciliary bodies compensate oxygen delivery to the retina, and the levels of hypoxia may vary across the peripheral avascular area in ROP. In this study, we have investigated a direct method for imaging gradient levels of retinal hypoxia at the peripheral avascular retina using a model ROP.

**Methods.:**

The rat 50/10 oxygen-induced retinopathy (OIR) model was generated by exposing the newly born Brown-Norway rat pups to a 24 hours alternate cycles of 50% and 10% oxygen for 14 days. We also confirmed the development of neovascularization in this model. HYPOX4 was used as a direct method for imaging gradient levels of retinal hypoxia at the peripheral avascular retina. A separate group of rat OIR pups were used to confirm gradient levels of retinal hypoxia using pimonidazole immunostaining. Gradient levels of retinal hypoxia was analyzed using ImageJ software from fluorescence intensities of HYPOX-4 and Pimonidazole immunostaining.

**Results:**

Retinal hypoxia was observed in the peripheral avascular retinas in rat OIR. Based on fluorescence intensity measurements, retinal hypoxia was at minimal levels near the ciliary bodies. Retinal hypoxia was at its maximum levels towards the avascular-vascular transition zones. Interestingly, we observed hemiretinal avascular retina temporal to the optic nerve in this OIR model, similar to human ROP retinas. In the retinal cross-section, hypoxia was not detectable near the ora serrata in rat OIR may be due to oxygen delivery by the ciliary bodies. Both pimonidazole and HYPOX-4 showed similar patterns of retinal hypoxia at the peripheral avascular retina in this model. As expected, preretinal neovascularization was observed at the avascular-vascular transition zones arising from the existing retinal vascular structures in this OIR model in Brown-Norway rats.

**Conclusions:**

In this study, we have characterized gradient levels of retinal hypoxia in the rat model of 50/10 OIR using a direct method from HYPOX-4 fluorescence. We observed minimal levels of retinal hypoxia near the ciliary bodies in this model and increased towards the avascular-vascular transition zones. In addition, we observed that the central vascularized retina remains gradient hypoxic in this model which could be detected using HYPOX-4. This study may clarify our understanding of retinal hypoxia in the ROP patient at the peripheral retinas.

## INTRODUCTION

Retinopathy of prematurity (ROP) is a leading cause of vision loss in premature infants and its pathogenesis has been described as consisting of two phases, Phase I and II (1, 2). Phase I culminates in an ischemia-induced retinal hypoxia (3–5). Preterm infants with an immature retinal vasculature are administered supplemental oxygen to compensate for underdeveloped lung function, which may cause systemic oxygen levels to rise periodically. However, due to systemic maladies such as patent ductus arteriosus (PDA) that are associated with prematurity and the necessary manipulations required for the care of the infant, episodes of low oxygen tension may also occur. Due to the combination of aforementioned as well as other treatments, conditions and events, the premature infant experiences variable oxygen levels throughout the course of oxygen treatment. Variable oxygen attenuates normal retinal vascular development, and when the oxygen therapy is discontinued, the infant is left with a large peripheral avascular retina (ischemia) that rapidly becomes hypoxic. Molecular studies have shown that retinal hypoxia increases the expression of proangiogenic growth factors and cytokines; the most important of these is vascular endothelial growth factor (VEGF)(6, 7). VEGF triggers the onset of the vasoproliferative phase (Phase II) of ROP, resulting in the formation of pre-retinal dysplastic structures commonly referred to as neovascular tufts (3, 8). These structures are leaky, fragile, prone to hemorrhage, predisposing the affected infant to tractional retinal detachment and blindness.

Upon considering the integral role of hypoxia in ROP pathogenesis, it becomes evident that a reliable non-invasive method for detecting, measuring and imaging retinal hypoxia in premature infants would offer great clinical utility. For example, infants could be screened for retinal hypoxia as a predictor of progression to phase II, perhaps guiding the clinician to initiate a prophylactic therapy. Assessment of retinal hypoxia may also indicate the severity of retinopathy and it could also be used as a benchmark to gauge the efficacy of therapy against established neovascular disease. Though, methods have been developed for the measurement of oxygen tension levels in tissues; these include nuclear magnetic resonance (5, 6), retinal oximetry (9), phosphorescence lifetime imaging (10), doppler optical coherence tomography (D-OCT) (11), and visible-light OCT (12). Their application has provided a clearer understanding of the vascular oxygen supply and metabolism in the retina, none of these imaging methods have been used successfully to measure retinal hypoxia. Pimonidazole-mediated immunohistochemistry is the most common method to study retinal hypoxia, but this technique is limited for its method of examination and not suitable for clinical *in vivo* applications (13).

Our laboratory has developed HYPOX-4, a hypoxia sensitive fluorescent molecular imaging probe to detect retinal hypoxia in the living retina (14–16). In the current study, we have investigated the application of HYPOX-4, as a direct method to detect and measure retinal hypoxia in the 50/10 oxygen induced retinopathy (OIR) using Brown-Norway rats. This model faithfully recapitulates several of the pathologic features of human ROP (17, 18). Previously, we have demonstrated the development of HYPOX-4 for the assessment of retinal hypoxia in mouse OIR (14), another model with an ischemia-induced hypoxia pathologic component. In this approach, the systemically administered HYPOX-4 is delivered successfully to the hypoxic avascular retina where it is presumably retained by the reduction of hypoxia-regulated nitro-reductases, thus allowing real time *in vivo* hypoxia-dependent fluorescence imaging (19). In the current study, we tried to overcome the challenges to deliver the HYPOX-4 after intraperitoneal injections to a remote peripheral avascular retina in the rat OIR model. We have used HYPOX-4 to characterize the levels and distribution of hypoxia in this rat 50/10 OIR model as a direct method to detect hypoxia in the peripheral retina. Furthermore, HYPOX-4-dependent imaging of gradient levels of retina hypoxia was compared to the profiles obtained using pimonidazole-adduct immunostaining method. Herein we report our results.

## MATERIALS AND METHODS

### Synthesis of HYPOX-4

**Figure 1:**
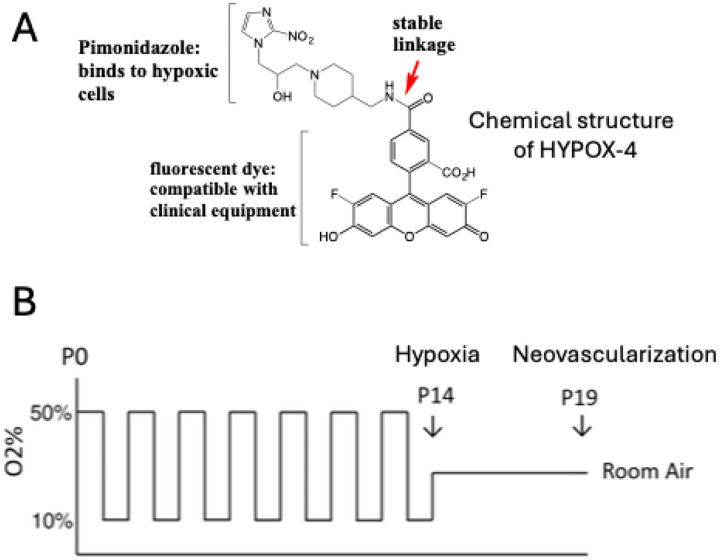
Direct method for imaging gradient levels of hypoxia in rat 50/10 oxygen-induced retinopathy (OIR) model. (A) Chemical structure of HYPOX-4. This in vivo imaging probe contains hypoxia sensitive compound, pimonidazole conjugated via an amide linkage to dye compatible with clinically used fluorescence imaging equipment. HYPOX-4 is water soluble and has no residual toxicity to the retinal cells. (B) Graph of inspired oxygen treatment to develop 50/10 OIR model in Brown Norway rat pups. In this model, newborn rat pups experience alternating episodes of 50% oxygen for 24 hours then 10% oxygen for 24 hours for a total of 14 days. At postnatal day-14 (P14) pups are returned to room air. Retinal hypoxia was monitored at P14. This model recapitulates features of neovascularization and intensifies at P19, similar to infants with severe ROP.

The HYPOX-4 was synthesized according to our previously reported methods(14). Chemical structure of HYPOX-4 is shown in Fig. 1. This in vivo molecular imaging probe contains a hypoxia sensitive, pimonidazole compound conjugated to a clinically compatible fluorescent dye via an amide linkage. HYPOX-4 is water soluble and has no residual toxicity to the retinal cells.

### Animals

Multi-timed pregnant Brown Norway Female rats were purchased from Charles River Laboratories; Chicago, Illinois. All animal procedures used in this study were approved by the Vanderbilt University Institutional Animal Care and Use Committee (Institutional approval number M1600260–01) and were performed in accordance with the ARVO Statement for the Use of Animals in Ophthalmic and Vision Research and in compliance with ARRIVE guidelines. Animals were group-housed according to their randomly assigned experimental groups in ventilated cages maintained under a 12 hours light and dark cycle at 22 ± 2°C within an institutional animal care facility. Animals were provided with clean water (Nashville Metro Water Services, Nashville, TN) and a standard diet consisting of 4.5% fat (PicoLab Rodent Diet 5L0D; LabDiet, St. Louis, MO) ad libitum. Rats were sacrificed by CO_2_-induced asphyxiation followed by cervical dislocation.

### Rat 50/10 oxygen-induced retinopathy model in Brown Norway

To generate Brown Norway rat 50/10 oxygen-induced retinopathy model (50/10 OIR), newborn rat pups were treated with alternating episodes of 50% oxygen for 24 hours then 10% oxygen for 24 hours for a total of 14 days. After the oxygen treatments, rat pups with their nursing mother return from the 50/10 OIR chamber to the normal room air. This treatment protocol mimics the variable systemic oxygen levels observed in cohorts of premature infants treated in intensive care units. At the end of the oxygen-treatment there is a peripheral avascular retina similar to that observed in human ROP whereas both are the exact opposite to the pattern observed in mouse OIR post oxygen-treatment.

### Direct method for imaging retinal hypoxia

HYPOX-4 was used as the direct method, and pimonidazole-adducts immunoassaying was used as indirect method to detect retinal hypoxia. After returning from the 50/10 OIR chamber to room air, the rat pups were intraperitoneally injected with 60 mg/kg HYPOX-4. Eighteen hours post-intraperitoneal injection, animals were sacrificed and retinas were dissected, and stained with isolectin B4 conjugated to AF647 to visualize the retinal vasculature by confocal microscopy. To evaluate the presence of hypoxia at the peripheral avascular retina, pimonidazole-adducts immunostaining method was used. Pimonidazole hydrochloride was injected intraperitoneally at a dose of 60 mg/Kg on day-14 (P14) at two hours after removal to room air. Two hours after the pimonidazole injection, animals were sacrificed. Retinas were dissected, immunoassayed for pimonidazole-adducts using antibody against pimonidazole-adducts (Hypoxyprobe, Burlington, MA, USA) was used to stain retinal hypoxia. ICAM-2 conjugated to AF-647 was used to counter stain for retinal blood vessels. In addition, retinal cross sections were also immunoassayed for pimonidazole-adducts. ImageJ software was used to quantify the levels of retinal hypoxia.

## RESULTS

### Direct method for imaging retinal hypoxia in rat 50/10 OIR using HYPOX-4

The rat 50/10 OIR model of ROP was developed in our laboratory to study the pathogenesis of neovascular retinopathy in premature infants (20–22). Among the models of ROP, it most closely recapitulates a majority of the pathologic features observed in human ROP patients. Among the disease pathologies, neovascularization is the most severe consequence of this disease condition. Levels of retinal hypoxia is an important indicator of severity of neovascularization. In this study, we have investigated the levels of retinal hypoxia in rat 50/10 OIR model. In this model, newborn rat pups were exposed to episodes of 50% oxygen for 24 hours then 10% oxygen for 24 hours for a total of 14 days (Fig. 1). At postnatal day 14 pups are returned to room air. Retinal hypoxia was monitored at P14.

**Figure 2:**
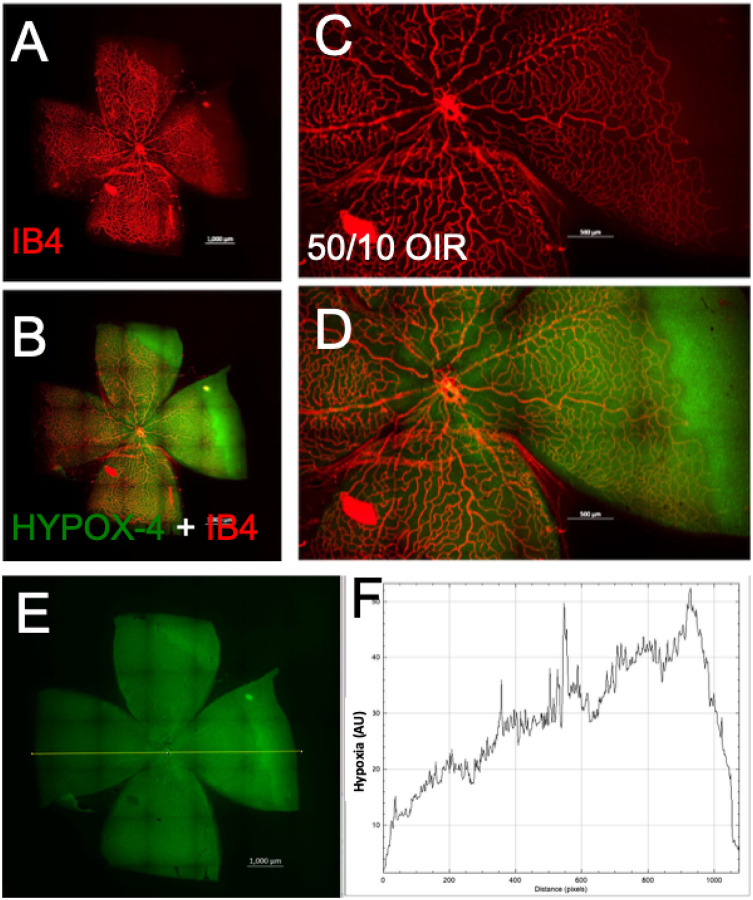
Imaging of retinal hypoxia using HYPOX-4 in 50/10 rat OIR. Isolectin B4 (IB4) was used to counter stain the vascular structures. (A, B) HYPOX-4 was localized largely in the avascular OIR retina. (C, D) Magnification of A and B respectively. (E, F) ImageJ software was used to analyze gradient levels of retinal hypoxia from HYPOX-4 fluorescence intensities across the 50/10 OIR retina as shown in F. Total of 12 eyes were analyzed for this study.

We used HYPOX-4 as a direct method for imaging gradient levels of retinal hypoxia in this model. Retinal hypoxia was observed in the peripheral avascular retinas in this model. Based on fluorescence intensity measurements, retinal hypoxia was at minimal levels near the ciliary bodies (Fig. 2). Retinal hypoxia was at its maximum levels towards the avascular-vascular transition zones. Interestingly, we observed hemiretinal avascular retina temporal to the optic nerve in this model, similar to human ROP retinas. In addition, we observed that the central retina is vascularized in this rat 50/10 OIR model; however the vascularized retina remains gradient hypoxic which could be detected using HYPOX-4 as shown in Fig. 2D–F. These observations may clarify the levels of retinal hypoxia in the ROP patient at the peripheral avascular retina and also at the vassalized areas of the ROP retinas.

### Characterization of retinal hypoxia in rat 50/10 OIR using pimonidazole-immunostaining

**Figure 3:**
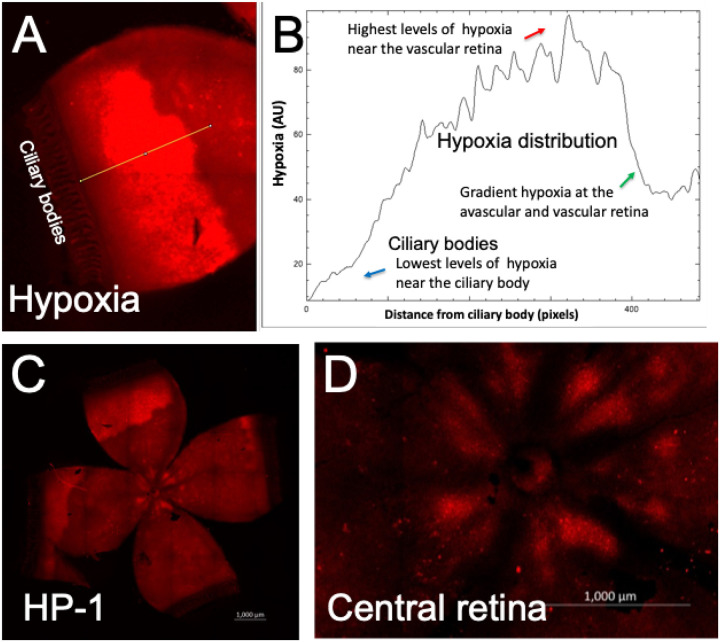
Retinal hypoxia was characterized in peripheral avascular retina in rat 50/10 OIR model. Pimonizadole hydrochloride was injected intraperitoneally at a dose of 60 mg/Kg on day-14, 2 hours after removal to room air. Rat pups were sacrificed two hours after the pimonidazole injection (which is four hours after removal to room air). Retinas were dissected, flat-mounted and immunoassayed for pimonidazole-adducts. (A, B) Gradient levels of retinal hypoxia were analyzed from fluorescence intensity measurements. ImageJ software was used to analyze the fluorescence intensities across the peripheral avascular 50/10 OIR retina. (C, D) Intense retinal hypoxia was observed at the peripheral avascular retina in this model. In addition, mild hypoxia was also observed at the central retina as shown in D. Total of 12 eyes were analyzed for this study.

We have further characterized retinal hypoxia at the peripheral avascular retinal using the standard pimonidazole-adduct immunostaining technique (Fig. 3). Retinal hypoxia was detected at the peripheral avascular retina. Gradient levels of retinal hypoxia were analyzed from fluorescence intensity measurements using ImageJ software (Fig. 3B). Both pimonidazole and HYPOX-4 showed similar patterns of retinal hypoxia at the peripheral avascular retina in this model. In addition, retinal hypoxia was observed at the vascularized area of the retina as shown in Fig. 3D. These results further confirmed the presence of retinal hypoxia even at the vascularized areas of the retina in this rat OIR model.

**Figure 4:**
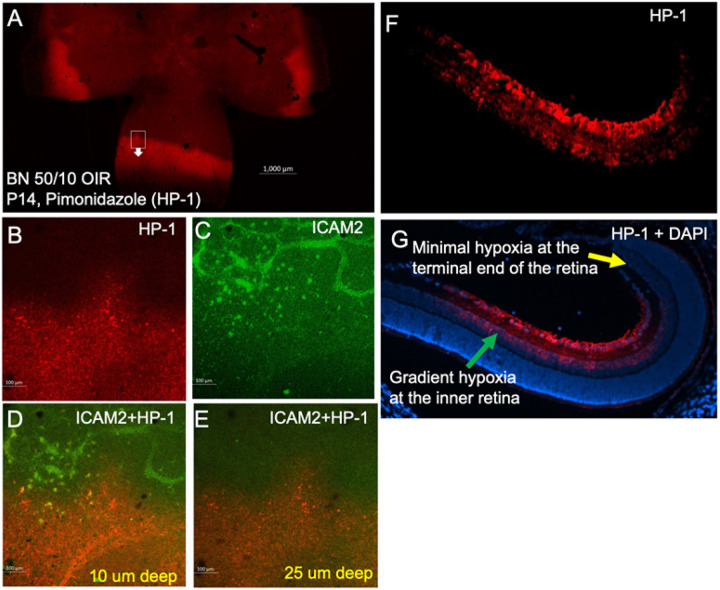
Imaging retinal hypoxia in 50/10 OIR retinal cross sections. Pimonidazole immunostaining (HP-1) was used to visualize the retinal hypoxia and ICAM-2 was used to counterstain retinal vascular structures. (A-E) Retinal hypoxia was observed at different depth of the 50/10 OIR retina, mostly in the inner retina. (F-G) Retinal hypoxia was also monitored in 50/10 rat retinal cross sections. Retinal hypoxia was observed at different layers including retinal ganglion cell layer (RGC), inner plexiform layer (IPL) and inner nuclear layer (INL). DAPI was used to counterstain the nuclei to localize HP-1 fluorescence in retinal layers.

**Figure 5:**
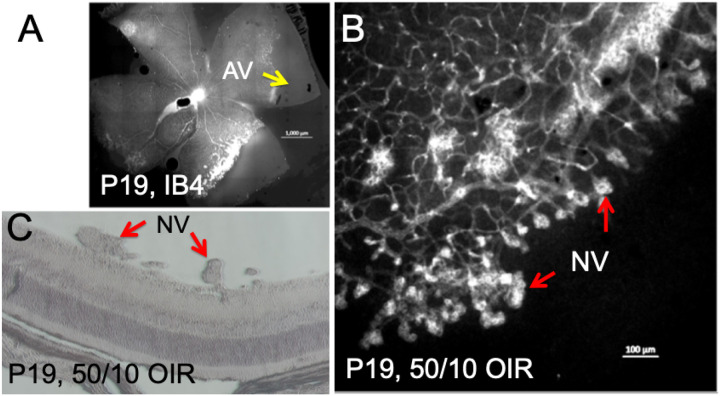
Characterization of preretinal neovascularization in Brown Norway 50/10 OIR model. (A) IB4 staining showing the neovascularization at the border of vascular/avascular area. Hypoxia may contribute to the development of neovascularization observed at P19. (B) Magnification view of A. (C) Preretinal neovascular tufts were localized in retinal cross section at P19.

In the retinal cross-section, hypoxia was not detectable near the ora serrata in the rat OIR model, may be due to oxygen delivery by the ciliary bodies (Fig. 4). In addition, retinal hypoxia was observed mostly at the inner retinal layers including retinal ganglion cell layer (RGC), inner plexiform layer (IPL) and inner nuclear layer (INL). As expected, preretinal neovascularization was observed at the avascular-vascular transition zones arising from the existing retinal vascular structures in this OIR model in Brown-Norway rats (Fig. 5). Thus, future clinical studies focusing on these findings could improve the diagnosis and treatment options for patients with hypoxic retinopathies.

## DISCUSSIONS

Retinal-hypoxia is associated with both early (phase I) and proliferative stage (phase II) of ROP, however the precise relationship between its onset, evolution and resolution, to other pathologic events, such as increased expression of proangiogenic growth factors and cytokines and ROP morphometrics (e.g. retinal vessel tortuosity, peripheral avascular area and severity of neovascular tuft formation etc.) is largely unknown (23). Therefore, the ability to reliably detect, measure and image retinal hypoxia would offer great advantages to the management of ROP. For example, infants could be screened for retinal hypoxia as a predictor for transition into phase II. Quantification of retinal hypoxia may help to establish ROP severity and could also be used as a benchmark to gauge the efficacy of therapy against neovascular disease. Furthermore, accurate measurement of retinal hypoxia would be of great benefit to the researcher investigating ROP pathogenesis in experimental models of ROP-like disease, leading to a better understanding of the role of hypoxia in ischemic retinopathies and the development of new drugs.

In summary hypoxia plays an integral role in ROP pathogenesis and currently its relationship to other events in the ROP pathogenic cascade is largely unknown. An understanding of this relationship would be of great benefit in the management of ROP. Current methods that are routinely used to measure levels of tissue oxygen tension suffer from drawbacks that limit their application to real time *in vivo* imaging of retinal hypoxia. Our newly developed direct imaging method using HYPOX-4 is safe, effective and reliable to detect retinal hypoxia to achieve these goals.

## CONCLUSION

In this study, we have characterized the gradient levels of retinal hypoxia in the peripheral avascular retina in rat 50/10 OIR model of ROP. We have utilized HYPOX-4 as a direct method to detect gradient levels of retinal hypoxia in this model. We found that HYPOX-4 is a clinically relevant molecular imaging probe to detect retinal hypoxia and could be used in future studies to improve the diagnosis and treatments for patients with hypoxic retinopathies.

## Data Availability

All data that support the results of this study are available within the article or upon request to the corresponding author (MIU).

## CLINICAL IMPLICATIONS

1. Imaging gradient levels of retinal hypoxia is an important indicator of severity of ROP pathogenesis including neovascularization. We used HYPOX-4 as a direct method for imaging gradient levels of retinal hypoxia in a model of ROP.
2. We observed minimal levels of retinal hypoxia near the ciliary bodies in this rat 50/10 OIR model and increased levels of hypoxia towards the avascular-vascular transition zones.
3. Even though the central area of the rat 50/10 OIR retina is vascularized, the vascularized retina remains gradient hypoxic which could be detected using HYPOX-4.
4. This study may clarify our understanding of levels of retinal hypoxia in the ROP patient at the peripheral avascular retina; and also, at the vascularized areas of the retina which is significant and needs future clinical investigations.

## Abbreviations

BM: basement membrane
CC: choriocapillaris
DCP: deep capillary plexus
FA: Fluorescein Angiography
GCL: ganglion cell layer
INL: inner nuclear layer
IPL: inner plexiform layer
LPS: Lipopolysaccharide
MCP: middle capillary plexus
NV: neovascularization
OCT: optical coherence tomography
ONH: optic nerve head
ONL: outer nuclear layer
OPL: outer plexiform layer
PDR: proliferative diabetic retinopathy
ROP: retinopathy of prematurity
SCP: superficial capillary plexus
VEGF: vascular endothelial growth factor
